# Effective management of pregnancy- and lactation-associated osteoporosis using romosozumab: a clinical case report

**DOI:** 10.1093/jbmrpl/ziaf141

**Published:** 2025-09-02

**Authors:** Atsushi Goshima, Hideki Tsuboi, Takashi Kaito, Motoki Iwasaki

**Affiliations:** Department of Orthopaedic Surgery, Osaka Rosai Hospital, Sakai 591-8025, Japan; Department of Orthopaedic Surgery, Osaka Rosai Hospital, Sakai 591-8025, Japan; Department of Orthopaedic Surgery, Osaka Rosai Hospital, Sakai 591-8025, Japan; Department of Orthopaedic Surgery, Osaka Rosai Hospital, Sakai 591-8025, Japan

**Keywords:** pregnancy, osteoporosis, breastfeeding, romosozumab, fracture

## Abstract

Pregnancy- and lactation-related osteoporosis (PLO) is a rare condition characterized by reduced bone density and increased fracture risk during late pregnancy or early postpartum. Because of its rarity, no well-established treatment exists for PLO. Here, we report a case demonstrating significant improvements in bone density and the absence of new fractures after 12 mo of romosozumab (ROMO) treatment following weaning. This report suggests that ROMO could effectively increase bone density and reduce fracture risk in patients with PLO.

## Graphical Abstract

**Graphical Abstract:**
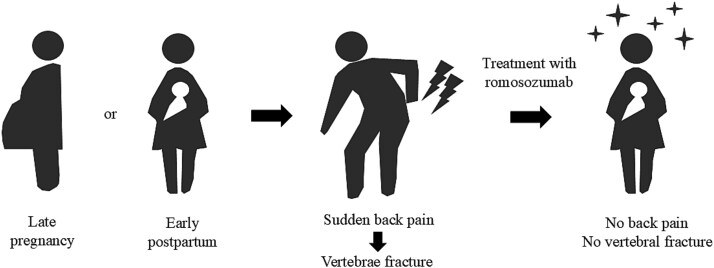

## Introduction

Pregnancy- and lactation-related osteoporosis (PLO) is a rare form of osteoporosis that primarily presents as fragility fractures during late pregnancy or early postpartum, most commonly affecting the vertebrae.^1^ Because of its rarity, no standardized treatment protocol exists for PLO; management strategies have included weaning, various medications, and exercise therapy.^2^ Here, we present the case of a patient with PLO who responded effectively to romosozumab administration.

## Case

**Figure 1:**
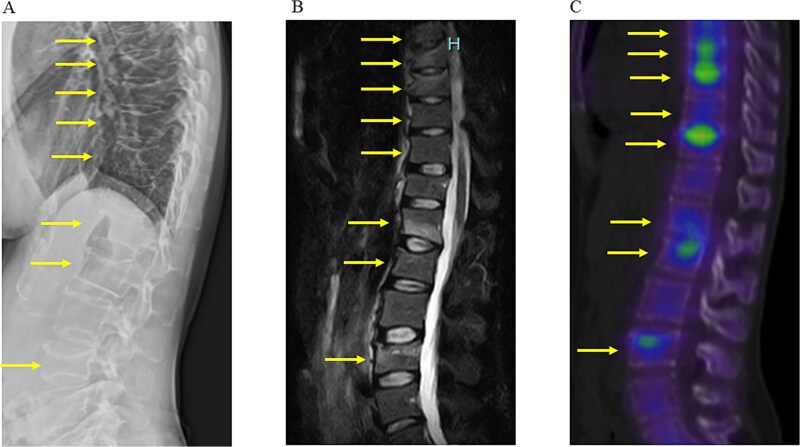
Pretreatment LS imaging. (A) Lateral radiograph of the vertebrae. (B) Lateral short tau inversion recovery (STIR) magnetic resonance imaging of the vertebrae. (C) ^99m^Tc-HMDP bone scan of the vertebrae. Arrow indicates the vertebral fracture.

A 34-yr-old woman presented with back pain that started 1 mo after delivering first child. Initial radiography at another clinic revealed multiple vertebral fractures, prompting her referral to our hospital for further evaluation and treatment after 2 mo. Her medical history was unremarkable, and she reported no alcohol or tobacco use and was not on any medications at the time. However, her family history was positive for osteoporosis, as both her mother and grandmother had received treatment. Physical measurements at our clinic indicated a height of 167 cm, weight of 50 kg, and BMI of 17.9 kg/m^2^. Further imaging confirmed multiple vertebral fractures at Th7, Th8, Th9, Th10, Th11, L1, L2, and L4 (Figure 1), with no evidence of pathological fractures, such as those due to metastatic disease. Table 1 summarizes serum laboratory data related to bone and mineral metabolism. Additional laboratory findings were as follows: thyroid-stimulating hormone 3.736 μU/mL (reference range: 0.541-4.2), free triiodothyronine 2.24 ng/dL (1.68-3.67), antinuclear antibody <40 (<40), rheumatoid factor 0 (<15 IU/mL), and anticyclic citrullinated peptide antibody <0.5 (<4.5 U/mL).

**Table 1:** Serum laboratory data related to bone and mineral metabolism.

Measurements	Before Treatment	6 mo	12 mo
**Ca (mmol/L) (normal range, 2.20-2.60)**	2.45	2.35	2.4
**P (mmol/L) (normal range, 0.80-1.45)**	1.2	0.95	0.95
**ALP (c) (normal range, 38-113)**	110	49	43
**PTH (pg/mL) (normal range, 10–65)**	19		
**TRACP-5b (mU/dL) (normal range, 120-420)**	668	142	44
**PINP (ng/mL) (normal range, 16.8-70.1)**	97.1	64.9	33.2
**25(OH)D (ng/mL) (normal range, 20-100)**	12.3		

**Figure 2:**
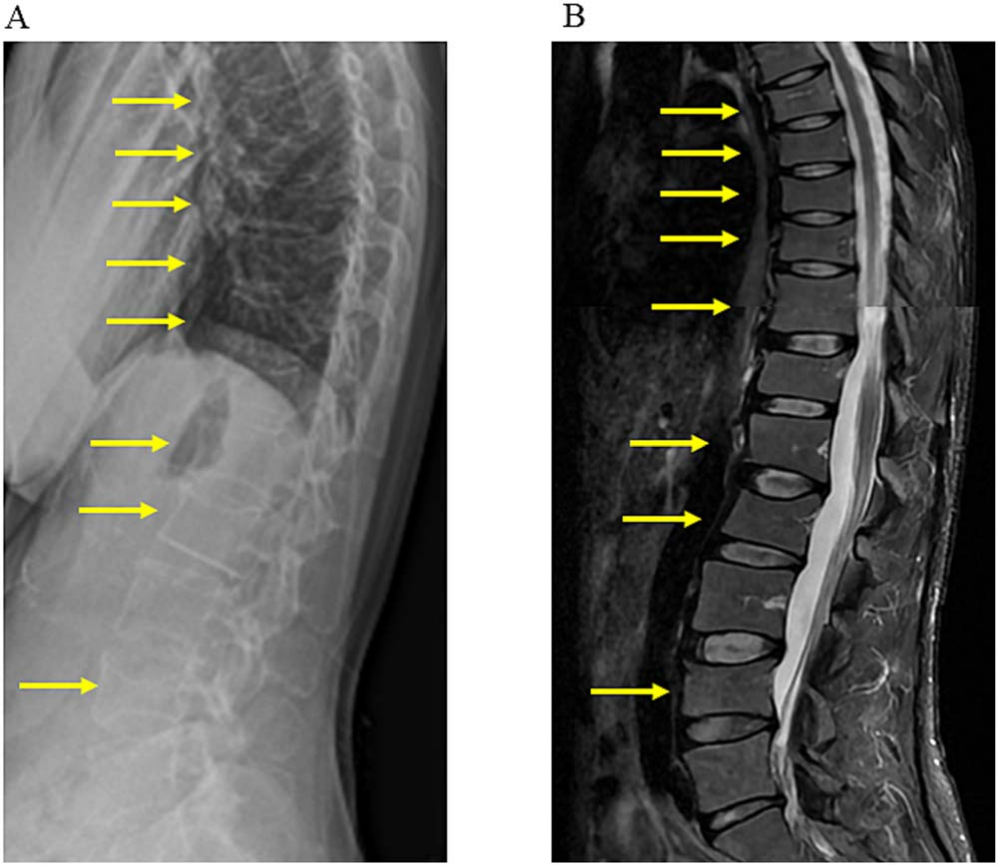
Lumbar spine imaging after treatment. (A) Lateral radiograph of the vertebrae. (B) Lateral STIR magnetic resonance imaging of the vertebrae. Arrows indicate vertebral bodies fractured before treatment. No progression of vertebral collapse or new vertebral fractures was observed.

Bone mineral density (BMD) was assessed by DXA. The BMD values and corresponding Z-scores were: LS: 0.702 g/cm^2^, Z-score: −3.0; right FN: 0.628 g/cm^2^, Z-score: −1.3; TH: 0.755 g/cm^2^, Z-score: −0.9. No evidence of secondary osteoporosis was noted; therefore, a diagnosis of PLO was established. Considering the high risk of additional fractures, treatment that included breastfeeding cessation, vitamin D3 and calcium supplementation, and romosozumab administration was initiated. After 1 yr of treatment, significant improvements were observed. In particular, we observed a 14.5% increase in LS BMD, 6.7% increase in FN BMD (Table 2), and a resolution of back pain, with no new vertebral fractures (Figure 2). However, her TH BMD decreased by 6.5% (Table 2). Although the importance of sequential therapy and a continuation plan was explained to the patient, she declined further treatment. The patient was recommended to continue taking vitamin D3 and calcium supplements to support bone health if she intended to become pregnant or give birth in the future. She returned to our hospital 19 mo after completing romosozumab treatment, following the birth of her second child. Her BMD values remained stable (Table 2).

**Table 2:** Changes in bone mineral density (BMD) before and after romosozumab administration.

		Before treatment	6 mo	12 mo	31 mo
**Lumbar spine**	BMD g/cm^2^	0.702	0.777	0.804	0.821
	Change from base line (%)		10.7	14.5	17.0
	Z-score	−3.0	−2.0	−1.7	−1.7
		**Before treatment**	**6 mo**	**12 mo**	**31 mo**
**Femoral neck**	BMD g/cm^2^	0.628	0.61	0.67	0.689
	Change from base line (%)		−2.9	6.7	9.7
	Z-score	−1.3	−1.4	−0.9	−0.6
		**Before treatment**	**6 mo**	**12 mo**	**31 mo**
**Total hip**	BMD g/cm^2^	0.755	0.682	0.706	0.71
	Change from base line (%)		−9.7	−6.5	−6.0
	Z-score	−0.9	−1.5	−1.3	−1.3

## Discussion

Pregnancy- and lactation-related osteoporosis, first reported in 1955 by Nordin et al.,^3^ has an estimated prevalence of 4-8 cases per million women.^4^ A recent analysis of Japanese national health insurance claims data estimated the incidence of PLO at approximately 460 cases per one million deliveries.^5^

Although the exact etiology of PLO remains unclear, some hypotheses indicate that this condition results from calcium depletion and hormonal changes that disrupt bone metabolism during pregnancy and lactation. Calcium is essential for fetal development, with fetal skeletal growth during pregnancy requiring approximately 30 g.^6^ To meet this demand, intestinal calcium absorption approximately doubles during pregnancy.^7^ However, inadequate calcium intake can trigger secondary hyperparathyroidism, thereby accelerating bone resorption. Moreover, during the third trimester, the breasts and placenta secrete large amounts of parathyroid hormone-related protein (PTHrP), which further promotes bone resorption.^7^ Although intestinal calcium absorption returns to baseline after delivery, the calcium needed for lactation is derived from maternal bones, thereby increasing bone resorption.

During postpartum, elevated prolactin levels stimulate milk production. High prolactin levels and ongoing lactation suppress the hypothalamic-pituitary-gonadal axis, subsequently inhibiting gonadotropin secretion and ovarian function.^7^ This inhibition reduces estradiol levels, which consequently increase osteoclast activity by upregulating RANKL and downregulating osteoprotegerin, leading to enhanced bone resorption and suppressed bone formation.^8^

Despite the plausibility of the proposed mechanisms, the precise pathophysiology remains unclear, and so various therapeutic strategies have been explored. Some reports suggest that BMD may recover spontaneously after weaning and return of menstruation. However, patients with multiple vertebral fractures are at a higher risk of subsequent fractures than those with single fractures, indicating a potential need for pharmacological intervention.^9^

Anagnostis et al. reported a 6.2% increase in LS BMD after 12 mo of vitamin D supplementation,^2^ suggesting its potential as a beneficial treatment option for patients with PLO. Nonetheless, most experts recommend considering alternative pharmacologic treatments in cases involving multiple vertebral fractures or persistent, disabling pain.^9^

Bisphosphonates, which inhibit osteoclast activity, have been considered effective in the treatment of PLO, with significant therapeutic outcomes having been reported.^10^ However, bisphosphonates accumulate in the bone and are slowly released, raising concerns about potential placental transfer and fetal exposure during future pregnancies.^11^ Hence, bisphosphonates are generally avoided in women planning to conceive within the following year.

Denosumab is a monoclonal antibody that targets RANKL, thereby blocking osteoclast activation and differentiation. This action suppresses bone resorption and increases BMD. A single 60-mg dose of denosumab has an average half-life of approximately 25.4 d, with serum levels gradually declining over 4-5 mo. Multiple reports have documented the effectiveness of denosumab in the treatment of patients with PLO.^12^

Despite its therapeutic efficacy, denosumab discontinuation has been associated with a rebound increase in bone turnover, commonly termed as an “overshoot.”^13^ To mitigate this rebound effect, sequential treatment with other antiresorptive agents is generally recommended after discontinuing denosumab.

However, the optimal management for patients desiring future pregnancies remains uncertain, presenting a clinical challenge in balancing therapeutic efficacy with reproductive planning.

Teriparatide has been considered a robust treatment option for patients with PLO. As a recombinant form of human parathyroid hormone (1-34), teriparatide has a short half-life of approximately 1 h, and its use in PLO has been documented in multiple case reports. Numerous studies have shown marked increases in BMD at the LS and FN following teriparatide therapy.^14^ In certain cases, improvements in BMD persisted even after completing a 1-yr course of therapy.^15^

However, preclinical studies have identified the development of osteosarcoma in rats treated with teriparatide.^16^ Although no such cases have been reported in humans, the duration of teriparatide treatment has been limited to 2 yr after considering these findings. Therefore, clinicians must carefully assess the risks and benefits when initiating teriparatide in practice.

Romosozumab is a monoclonal antibody that targets sclerostin, a glycoprotein that inhibits bone formation by antagonizing the Wnt/β-catenin signaling pathway. It exerts a dual action by promoting bone formation and suppressing bone resorption, which substantially increases BMD.^17^

To date, only one case has reported the use of romosozumab for the treatment of PLO.^18^ In this particular case, romosozumab was selected because of the patient’s intolerance to teriparatide, which promoted a notable improvement in BMD. The ARCH trial indicated an increased incidence of cardiovascular events with romosozumab^19^; however, the FRAME trial found no such association.^20^ Nonetheless, potential cardiovascular risks should be accounted for when considering its use.

In the present case, the patient developed multiple vertebral fractures, which necessitating a treatment that could rapidly increase BMD. Furthermore, because the patient planned to conceive again, a treatment that could potentially be re-administered in the future was needed. Considering these clinical factors, romosozumab was selected as the treatment of choice.

Although an initial increase in PINP, a marker of bone formation, is expected following romosozumab administration, its levels progressively decline over time. This decrease has also been reported in previous studies. Although the exact mechanism remains unclear, potential contributing factors include hormonal influences, such as estrogen and PTHrP, as well as the pharmacological action of the drug itself. Nevertheless, the observed changes remained within the normal range and were not considered clinically significant. Furthermore, a decrease in total femoral BMD from baseline was noted, the cause of which remains unidentified.

In the present case report, we showed that romosozumab significantly increased BMD and prevented recurrent fractures in a patient with PLO who presented with multiple vertebral fractures. Moreover, BMD was maintained even after romosozumab discontinuation and a subsequent pregnancy and delivery, with no new fractures observed. To the best of our knowledge, this is the first report on the use of romosozumab for the treatment of PLO.

Despite ongoing concerns about cardiovascular risks, cost, and potential fetal effects, romosozumab may be a promising treatment option for patients with PLO and multiple vertebral fractures.

## Data Availability

The data cannot be shared publicly due to privacy issues. The patient has provided her consent for this case report.
